# The disappearing northern leopard frog (*Lithobates pipiens*): conservation genetics and implications for remnant populations in western Nevada

**DOI:** 10.1002/ece3.308

**Published:** 2012-07-22

**Authors:** Serena D Rogers, Mary M Peacock

**Affiliations:** 1 Department of Biology, University of NevadaReno, Nevada, 89557; 2 Alaska Department of Fish and GameAnchorage, Alaska, 99518-1599

**Keywords:** Conservation, microsatellite DNA, mitochondrial DNA, northern leopard frog, remnant populations

## Abstract

Global amphibian declines suggest a major shift in the amount and quality of habitat for these sensitive taxa. Many species that were once widespread are now experiencing declines either in part of or across their historic range. The northern leopard frog (*Rana* [*Lithobates*] *pipiens*] has undergone significant declines particularly in the western United States and Canada. Leopard frog population losses in Nevada are largely due to habitat fragmentation and the introduction of nonnative fish, amphibian, and plant species. Only two populations remain in the Truckee and Carson River watersheds of western Nevada which represents the western boundary of this species range. We used sequence data for an 812 base pair fragment of the mitochondrial NADH dehydrogenase 1 (ND1) gene to support a native origin for western Nevada populations. All frogs had a single haplotype (W07) from the distinct western North America ND1 haplotype clade. Data from seven polymorphic microsatellite loci show that Truckee and Carson River populations are highly differentiated from each other and from leopard frogs collected from eastern Nevada sites. Lack of gene flow among and distinct color morphs among the western Nevada populations likely predates the current geographical isolation. Comparisons with other peripheral *L. pipiens* populations show western Nevada populations have similar levels of gene diversity despite their contemporary isolation (*H*_E_ 0.411, 0.482). Restoration of leopard frog populations in these watersheds will be challenging given well-entrenched nonnative bullfrog populations and major changes to the riparian zone over the past century. Declines of once common amphibian species has become a major conservation concern. Contemporary isolation of populations on a species range periphery such as the leopard frog populations in the Truckee and Carson rivers further exacerbate extirpation risk as these populations are likely to have fewer genetic resources to adaptively respond to rapidly changing biotic and abiotic environments.

## Introduction

Global amphibian declines were first documented in the 1980s and more than 500 amphibian populations were designated “of concern” by 1993 (Blaustein and Kiesecker 2002). By 2008, one-third of the approximately 6347 described amphibian species were considered in decline (Collins and Crump 2009; Crump 2009). Wake and Vredenburg (2008) suggest that amphibians, as a group, are at risk of global extinction and may be early indicators of a sixth mass extinction. Habitat degradation and destruction have been widely implicated in amphibian decline (Blaustein et al. 1994; Hecnar 1997; Stromberg et al. 2004; Nystrom et al. 2007), with additional causal mechanisms including chemical contaminants (Bridges and Semlitsch 2000; Hatch and Blaustein 2003; Relyea 2003), ultraviolet-B (UV-B) radiation (Blaustein et al. 2003), disease (especially Chytridiomycosis) (Daszak et al. 2003; Briggs et al. 2005), overexploitation, (Jennings and Hayes 1985), and the introduction of nonnative species (Hayes and Jennings 1986; Kiesecker et al. 2001; Matthews et al. 2001; Kats and Ferrer 2003). Because amphibians play important roles in ecosystems as herbivores, predators, and prey, population losses will likely have large implications for ecosystem processes (Blaustein et al. 1994).

**Figure 1:**
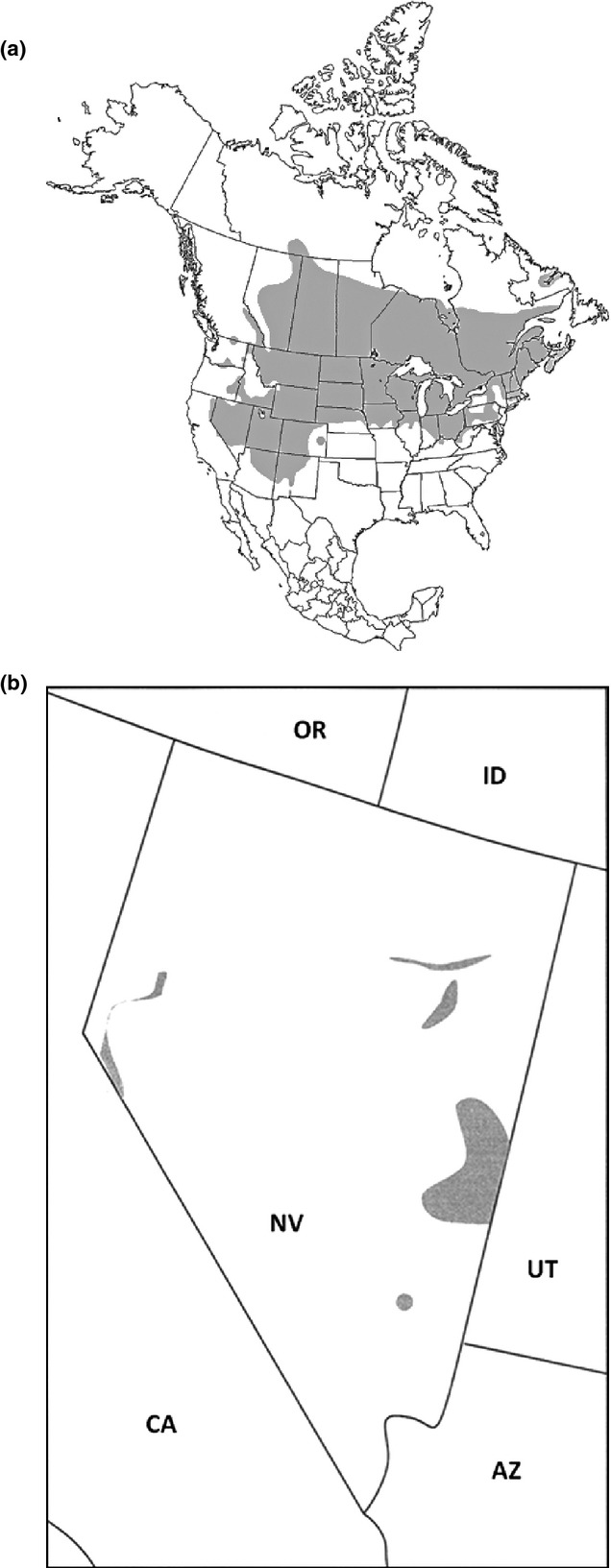
(a) Historical range of *Lithobates pipiens* (Smith and Keinath 2007) and (b) current distribution of *L. pipiens* in Nevada (reprinted with permission from C. Hitchcock).

**Figure 2:**
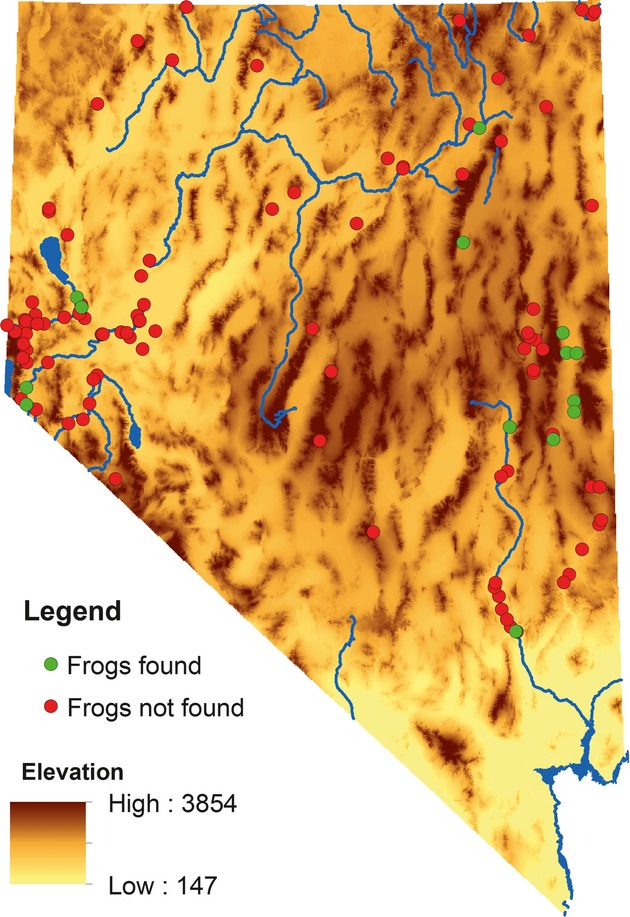
Historic and random sampling locations for leopard frogs in Nevada (Hitchcock (2001). *L. pipiens* was found at sites indicated with green-filled circles. Red-filled circles represent unsuccessful searches Map created by Joseph Stewart.

The northern leopard frog (*Rana* [*Lithobates*] *pipiens*; *L. pipiens* from this point forward) is widely distributed across North America (Fig. 1a) and is currently considered globally secure. However, the range map is deceiving as significant declines are occurring in the western United States and Canada, where once large leopard frog populations could be found (Leonard et al. 1999; Lannoo 2005). In some areas, *L. pipiens* has disappeared completely (Corn and Fogleman 1984; Bull and Wales 2001; Werner 2003). Surveys of the contemporary distribution of leopard frogs in Nevada suggest significant population losses statewide over the last 70 years (Fig. 1b, from Hitchcock 2001). Hitchcock's (2001) resurvey of historically occupied sites across Nevada revealed leopard frogs at only 18 of 97 sites (Fig. 2, from Hitchcock 2001) with populations largely extirpated from the north-central and northwestern portions of the state. In western Nevada, leopard frogs were once common along the Truckee, Carson, and Walker rivers; however, recent surveys have found only four occupied locations within these three watersheds. The origin of the extant frogs in the Truckee River is of particular interest as leopard frogs were introduced in the early 1900s from unknown locations to sites around Lake Tahoe in the upper Truckee River watershed as a supply for restaurants (Fig. 2; Bury and Luckenbach 1976). In Nevada, most of the extant leopard frog populations are found in eastern part of the state and appear to be completely isolated from the two currently occupied sites in western Nevada, as no frogs were found in intervening sites sampled along the Humboldt River that connects eastern and western watersheds (Fig. 2; Hitchcock 2001). Several populations in eastern Nevada were found in wetland areas in valleys which drain into the eastern reaches of the Humboldt River, but most occupied sites were in valleys to the south and east of the main stem river (Fig. 2).

Currently, the species is on the Nevada Natural Heritage Program “Watch List,” and United States Forest Service and Bureau of Land Management (BLM) have also designated the northern leopard frog as a sensitive species. Most recently, the United States Fish and Wildlife Service issued a 90-day finding to list the western populations of northern leopard frogs as threatened under the United States Endangered Species Act (United States Fish and Wildlife Service 2009).

A survey of locations historically occupied by leopard frogs (Hitchcock 2001) suggests that this species may be disappearing from Nevada with the remaining populations isolated and at risk for accelerated loss of genetic resources. Population isolation can lead to fitness declines which can occur through genetic bottlenecks, inbreeding, and subsequent inbreeding depression, ultimately leading to losses of genetic variation, fixation of deleterious alleles, and increases in genetic load (Beebee and Rowe 2008). Beebee and Rowe (2008) found decreased rates of metamorphosis and lower survival rates with an increased genetic load suggesting an association in small isolated populations of the natterjack toad (*Bufo calamita*). Although it is tempting to predict that individuals with high heterozygosity levels at neutral markers, such as microsatellites, will also have greater relative fitness, we recognize that such an association is far from certain. There is, however, some support for this prediction in the literature (Olano-Marin et al. 2011; Tamukai et al. 2011; Wetzel et al. 2012). Olano-Marin et al. (2011) compared heterozygosities for microsatellite loci linked to functional genes (*N* = 58 markers) and for neutral markers (*N* = 21) with clutch size and number of eggs produced by males in the Blue tit (*Cyanistes caeruleus*) and found a clear relationship between heterozygosity at the neutral markers and reproductive success, but not for microsatellites classified as “functional.” Wetzel et al. (2012) showed that heterozygosity at 21 neutral microsatellite in female house sparrows *(Passer domesticus*) was associated with reproductive performance (clutch size, egg size, hatching success, and nestling survival). These authors interpret the result of significant heterozygosity-fitness correlations as an indication that the effects of heterozygosity may be far more significant than previously thought (Wetzel et al. 2012). Although heterozygosity at major histocompatibility complex (MHC) loci, but not neutral loci, was positively associated with reproductive success in house mice (*Mus musculus*), Tamukai et al. (2011) found that effects of MHC heterozygosity depended to a large extent upon the heterozygosity of the background (neutral) genes such that maximal MHC heterozygosity was most beneficial at intermediate or optimal levels of background heterozygosity. Although we did not measure fitness-related characters, we address viability of the western Nevada leopard frog populations through analysis of population isolation, gene diversity, effective population size, and inbreeding using levels of genetic variation at neutral microsatellite markers. We base this approach upon the premise that neutral genetic variation can give some indication of levels of inbreeding and genetic resources within these populations. We also compare sequence data from mitochondrial DNA (mtDNA) ND1 gene for frogs from the extant Nevada populations to published sequences in order to assess whether leopard frogs in Truckee River basin were part of the distinct western ND1 haplotype clade. We focused primarily on the western Nevada populations as declines in this portion of the range are pronounced and leopard frogs appear in danger of local extirpation with the potential loss of private alleles and rare genotypes. While collecting tissue samples in the field, we noticed that there was a striking color difference between the Carson and Truckee River leopard frog populations. An additional question emerged as to whether phenotypic variation would correlate with genotypic variation between the Truckee River and Carson River populations.

## Methods

### Northern leopard frog (*Lithobates pipiens*)

**Figure 3:**
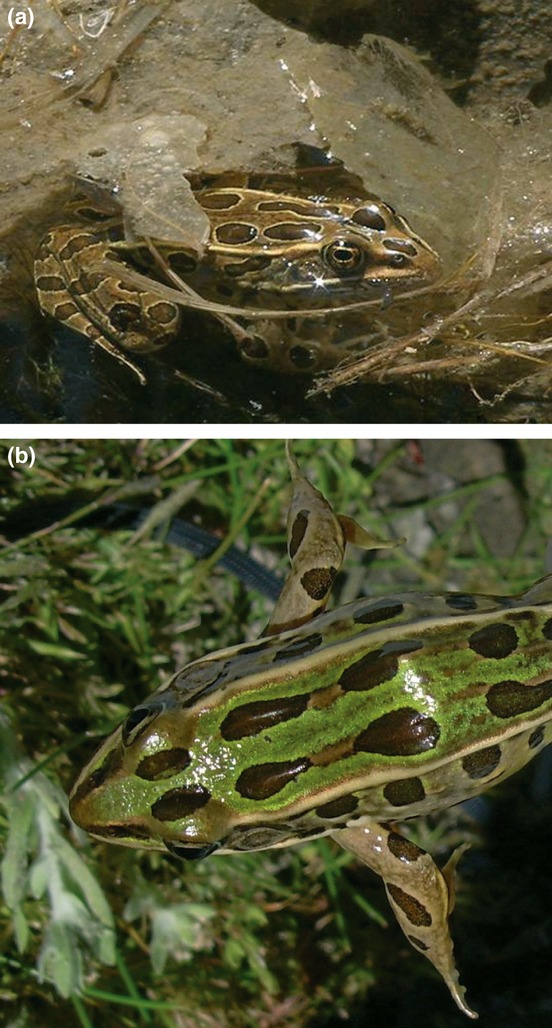
(a) Brown color morph (specimen from Truckee River, Nevada) and (b) green color morph (specimen from Carson River, Nevada). Photographs taken by S. Rogers.

Northern leopard frogs (*Rana* [*Lithobates*] *pipiens* Schreber, 1782) are characterized by dark spots on their dorsal side and dorsolateral folds. These frogs are considered medium sized and are typically green or brown (wild types; Fig. 3), but also occur with no spots (burnsi variation) or mottled spots (kandyohi) (Moore 1942; Volpe 1955; Merrell 1965). The green and brown coloration is controlled at a single locus at which the green allele is dominant (Fogleman et al. 1980). Although, both color morphs can co-occur, green morphs are more common in forest habitats (Moore 1942; Volpe 1955). The snout-to-vent length of most adult leopard frogs ranges from 5 to 10 cm with the females being larger than the males (Seburn and Seburn 1998). Dole (1965) found that adult home ranges are small ranging from 15 to 600 m^2^ and may include breeding sites, foraging area in the upland, dispersal corridors, and hibernacula (Merrell 1970). Migrations to and from upland and breeding sites within the leopard frog home range occur seasonally. During the summer, *L. pipiens* forage in habitats with adequate moisture to prevent desiccation, and during the winter, the frogs hibernate in areas that have some flowing water that prevent anoxic conditions (Hill 1981).

### Nevada northern leopard frog distribution and status

Leopard frogs were once reported as the most widespread amphibian species in the state (Linsdale 1940). Despite many historical localities in western Nevada, leopard frogs have recently only been found at four locations in the Truckee and Carson River watersheds (Hitchcock 2001; see below). Panik and Barrett (1994) found leopard frogs at a single site among 31 sites surveyed along the Truckee River, while Hitchcock (2001) found frogs at both the Panik and Barrett (1994) site and an additional site several kilometers north. The Panik and Barrett (1994) site was resurveyed for this study but no frogs were found. Although Hitchcock (2001) found two occupied sites in the Carson River drainage, a single, small, and isolated population remains in the watershed. Statewide, leopard frogs were found at only 18 of 97 sites where they were found historically (Hitchcock 2001). The majority of sites (*N* = 9) with Leopard frogs were found in the Spring and Lake valleys of eastern Nevada (Fig. 2).

### Study sites

**Figure 4:**
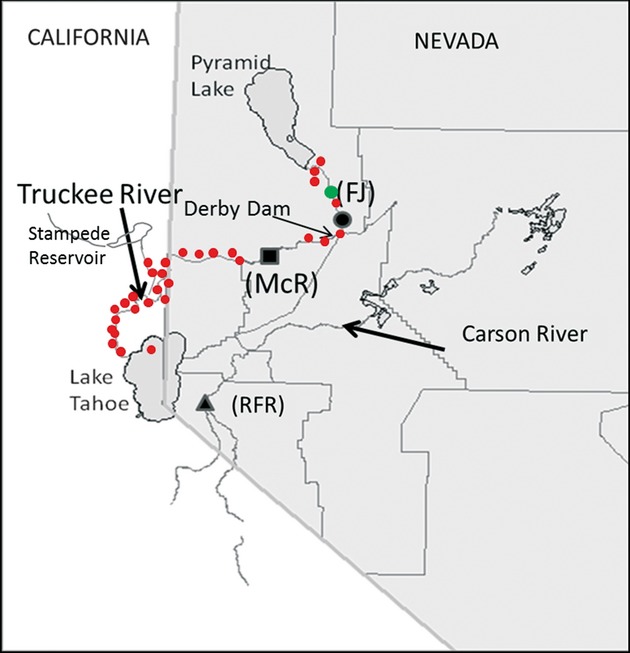
Map of western Nevada showing the Truckee and Carson rivers. Small red-filled circles (*N* = 31) are sites sampled by Panik and Barrett (1994). The small green circle is the site where both Panik and Barrett (1994) and Hitchcock (2001) found leopard frogs. The three sampling areas for this study are indicated by the largest circle, Paiute Indian Tribe property (FJ); square, McCarran Ranch (McR); triangle (RFR). Map was made using ArcMap in ArcGIS 93 by S. Rogers.

We used Hitchcock (2001) and Panik and Barrett (1994) to locate the sites in western Nevada that were sampled for this study (Figs. 2, 4). Eighteen samples collected by C. Hitchcock (2001); 16 eastern Nevada, 2 southern Idaho) were obtained from the museum collections at California State University, Northridge, for inclusion in this study.

#### Truckee River watershed

The Truckee River flows ∼193 km from Lake Tahoe in the Sierra Nevada mountain range to terminal Pyramid Lake. Truckee River riparian habitats have been significantly altered since the early 1900s. Installation of Derby Dam in 1905 and subsequent channelization of the lower Truckee River in 1962 by the Army Corps of Engineers (USACE) resulted in changes to the natural hydrology of the lower river (Fig. 4; Stromberg et al. 2004). Derby Dam was built to divert water via the Truckee Canal for agricultural uses in the Carson River watershed, which has resulted in a drastic reduction in flows in the lower Truckee River. Channelization of the river as a result of the Flood Control Act of 1954 caused extensive erosion in the riverbed and increased flooding downstream from the channelized areas. Alterations to seasonal flooding patterns and reduced natural recruitment in the riparian plant community have led to extensive declines in the wildlife populations that depend on river habitats (Rood et al. 2003). Changes to the riparian corridor are one of the main causes for the absence of leopard frogs along most of the lower river with the loss of Cottonwood (*Populus fremontii*) gallery forests and extensive invasion of tall whitetop (*Lepidium latifolium* L.) together with the introduction of nonnative amphibian species such as the North American bullfrog (*L. catesbeiana*). The only extant native population on the Truckee River located for this study was found on a private ranch near Wadsworth, Nevada on the Pyramid Lake Paiute Tribe Reservation, approximately 33 miles northeast of Reno, Nevada, which was also occupied during the Hitchcock survey (Fig. 4; FJ; Hitchcock 2001). Fifty-six adult frogs were sampled from the man-made ponds on the ranch, which are used primarily as watering holes for livestock. Leopard frog egg masses have been collected annually from this site to be raised and released at McCarran Ranch (McR), a Nature Conservancy restoration site, in an attempt to provide a source population for reintroduction of frogs to other areas within the Truckee River watershed.

The Nature Conservancy (TNC) has purchased multiple properties along the Truckee River for restoration activities aimed at returning the river to its natural course and function. TNC bought the 305 acre McR on the lower Truckee River approximately 15 miles east of Reno, Nevada, in 2002. Here, they restored a natural channel meander, created ponds, and backwater habitats. Frogs that developed from egg masses collected from a natural population on the Pyramid Lake Paiute Tribe Reservation and reared in captivity were released on the McR site from 2006 to 2009. The eight ponds at McR were extensively searched during this study; however, only four adult leopard frogs were found at a single pond location (Fig. 4) due largely to the high densities of invasive bullfrogs. Therefore, 30 tissue samples were taken from a captive population (CA) also founded with frogs from the FJ site, which is maintained by Otis Bay Ecological Consulting Firm (http://www.otisbay.com).

#### Carson River watershed

The Carson River is approximately 211 km long, originating in the Sierra Nevada of northern California and emptying into the Carson Sink in western Nevada (Fig. 4). The Carson River also underwent extensive channelization in the 1960s that resulted in channel erosion, sediment loading, and increased channel depths. In 1990, the Carson River was designated a Superfund site by the United States Environmental Protection Agency (EPA) due to mercury contamination (http://www.epa.gov) resulting from the Comstock Mining era in the 1860s to 1880s. Mercury deposited atmospherically into wetlands has been shown to cause severe deformities and mortality in developing southern leopard frogs (*L. sphenocephala*) (Unrine et al. 2004). As a result, the increased levels of mercury occurring in the Carson River raises concern for the population of leopard frogs found there along with other amphibian species.

Currently, the Carson Water Subconservancy District (http://www.cwsd.org) is working with multiple governmental and private agencies to improve the conditions of the Carson River, including a comprehensive stewardship plan encompassing the entire Carson River drainage. TNC has become involved in restoring a portion of the river at their 805 acre property, River Fork Ranch (RFR). Sixty-one frogs were collected along the banks of the river and small side channels at this site (Fig. 4).

#### Eastern Nevada

Hitchcock (2001) conducted an extensive survey of both historical locations that had known frog populations and an additional 20 wetland sites selected randomly from throughout the leopard frog range in Nevada that were not included in the historical record. We obtained toe clips from 18 vouchered specimens collected by C. Hitchcock in eastern Nevada (*N* = 16) and southern Idaho (*N* = 2) that were deposited in the museum collection at California State University, Northridge (Table 1). The average number of frogs observed by Hitchcock (2001) at sites surveyed in eastern Nevada ranged from 1 to 37 with fewer than five individuals observed at most locations. A single location, Ferguson Spring near Wendover, Nevada, has a robust population with ∼100 individuals observed during sampling. However, because of the presence of numerous springs and connecting habitat, there is likely a network of leopard frog populations throughout these eastern Nevada valleys (Hitchcock 2001). As the sample size from eastern Nevada was limited and collected from multiple locations, we could not conduct population level analyses for these samples, but we did assess nuclear microsatellite loci allelic richness and mtDNA sequence diversity for comparison with the western Nevada samples. The two Idaho samples were included in the mtDNA phylogenetic analysis only.

**Table 1:** *Lithobates pipiens* samples collected by C. Hitchcock in eastern Nevada and southern Idaho obtained from the California State University, Northridge collection

Catalog #	Date collected	Country	State	County	Latitude	Longitude	UTM coordinates
7419	25 June 2000	USA	Nevada	Elko	40.17216	–115.48061	
7531	3 July 2001	USA	Idaho	Caribou			UTM 413602 E; 4745880 N
7533	3 July 2001	USA	Idaho	Caribou			UTM 412375 E; 4748364 N
7535	30 June 2001	USA	Nevada	White Pine			UTM 0723684 E; 4304372N
7536	1 July 2001	USA	Nevada	White Pine			UTM 0723576 E; 4313366 N
7537	3 July 2001	USA	Nevada	Elko			UTM 0738744 E; 4479349 N
7538	3 July 2001	USA	Nevada	Elko			UTM 0738744 E; 4479349 N
7573	16 June 2001	USA	Nevada	Lincoln			UTM 0673349 E; 4117977 N
7582	10 July 2001	USA	Nevada	White Pine			UTM 0717279 E; 4354319 N
7583	11 July 2001	USA	Nevada	White Pine			UTM 0725682 E; 4354008 N
7584	12 July 2001	USA	Nevada	Lincoln			UTM 0706117 E; 4280825 N
7585	11 July 2001	USA	Nevada	White Pine			
7586	11 July 2001	USA	Nevada	White Pine			
7587	12 July 2001	USA	Nevada	White Pine			UTM 0706071 E; 4279970 N
7588	11 July 2001	USA	Nevada	White Pine			
7602	14 August 2000	USA	Nevada	Elko	40.42987	-114.18398	
7637	22 July 2001	USA	Nevada	Elko			UTM 0643055 E; 4545665 N
7639	22 July 2001	USA	Nevada	Elko			UTM 0643055 E; 4545665 N

Sampling date, location, and catalog number are listed.

### Sample collection

Visual encounter surveys were conducted along the edges of ponds, streams, and rivers within each of the study sites where *L. pipiens* habitat was abundant. Leopard frogs were typically located either floating along the edge of a body of water or in grassy vegetation growing in wet soil near a body of water. The FJ site consisted of three man-made ponds and various areas of the river's edge where the property owner had encountered leopard frogs. The man-made ponds within the FJ site are the only areas that leopard frogs were located and sampled. The RFR study site contains a two mile section of the Carson River. The edges of the main stem and streams most extensively searched were those where the banks of the river were even with the water's edge. The McR site consisted of eight ponds that were extensively searched. The edge of the Truckee River at this site was not searched due to suboptimal habitat. Leopard frogs were captured either by hand or dip-net. As recommended by the National Wildlife Health Center (http://www.nwhc.usgs.gov), frogs were held around the waist with hind limbs fully extended to prevent unnecessary struggling that could result in harm to the animal. Weight, snout-to-vent length, sex, and age were recorded for each individual along with Universal Transverse Mercator (UTM) coordinates from a global positioning system or latitude–longitude coordinates from United States Geological Survey 7.5-min series topographical maps of the area. A photograph was taken of each individual frog while they were being held as an additional aid in identification during data entry and in an attempt to have comparable images among frogs later used in the spot and color polymorphism analysis.

Guidelines from Leyse et al. (2003) were used for the tissue collection. Three to four millimeters of a single toe from the front and back feet were collected from each individual sampled. After toes were cut, the wound was sprayed with Bactine® (Martin and Hong 1991), and individuals were released onto land to prevent the medication from being immediately washed away by water. The toes that were sampled varied among individuals and sites to permanently mark each individual and ensure resampling of individuals did not occur. Toes were cut using surgical grade scissors (Fischer Scientific, catalog number 08-940). Each sample was stored in a coin envelope to allow for complete drying of the tissue. After each toe was removed, the scissors and tweezers/forceps were stored in hydrogen peroxide to avoid cross-contamination of samples and possible disease transfer among individuals. Prior to use, the scissors were rinsed with water, and completely dried with paper towels. Nitrile gloves were worn to sample each individual in order to avoid contaminating frogs with tissues or blood from previous individuals and to avoid contamination of the tissue samples. After sampling all frogs were released at their capture location. All animal handling was conducted in accordance with Institutional Animal Care and Use guidelines as, well as those of the American Society of Ichthyologists and Herpetologists.

### Mitochondrial DNA PCR protocol

DNA was extracted from toe clippings using a DNeasy Blood and Tissue Extraction Kit (Qiagen, Valencia, California) according to the manufacturer's specifications and then quantified using a Labsystems Fluoroskan Ascent fluorometer. An 812 base pair fragment of the mtDNA NADH dehydrogenase subunit 1 (ND1) gene was amplified using polymerase chain reaction (PCR). We used the MB77 forward (Hoffman and Blouin 2004a) and RpND1R reverse primers (Wilson et al. 2008) for eight individuals from the Pyramid Lake Paiute Reservation population (FJ), four from the McR site, three from the Carson River (RFR) population, 14 individuals from the eastern Nevada sites (with sufficient DNA), and two from southern Idaho. PCR was carried out in 12.5 *μ*L reaction volumes containing 1.25 *μ*L of each primer, 5 *μ*L 2× Qiagen master mix with *taq* polymerase, and 5 *μ*L of 15–20 ng/*μ*L genomic DNA. PCR cycle parameters consisted of an initial denaturation at 95°C for 15 min, followed by 35 cycles of denaturing at 94°C for 30 sec, annealing at 54°C for 90 sec, and a final 90-sec extension at 72°C. PCR product was purified using ExoSAP-IT (USB, Cleveland, Ohio) and then sequenced in both directions on an ABI 3730 DNA Analyzer, Nevada Genomics Center (http://www.ag.unr.edu/genomics), using the ABI BigDye Terminator Cycle Sequencing Ready Reaction Kit v3.1 (Applied Biosystems, Carlsbad, California).

### Microsatellite PCR protocol

Seven nuclear microsatellite loci were used for this study (Table 2; Hoffman et al. 2003; Monson and Blouin 2003; Hoffman and Blouin 2004b; Richter and Broughton 2005). Forward primers for five loci were constructed with one of three M13 tails (FAM, VIC, or NED), and corresponding reverse primers were “pigtailed” by adding (gtttcttt) to the 5′ end (Table 2) to allow attachment of the matching fluorescently labeled M13 primers in a second PCR (Schuelke 2000). Forward primers for two loci (Rpi107 and Rp193) were ordered fluorescently labeled with no modifications to the corresponding reverse primers. Loci were grouped by size into three panels to allow for multiplexing during PCR. Amplification was completed in two separate multiplex PCRs in MBS Satellite thermocyclers for the five-tailed primers (Table 2; panels A and C). The initial multiplex PCR for panels A and C contained 8 *μ*L Multiplex PCR Master Mix (Qiagen), 0.05 *μ*mol/L of each tailed forward primer and 0.5 *μ*mol/L reverse primers, and 20 ng DNA in a 16 *μ*L reaction volume. The first PCR was a touchdown allowing for complete amplification of the DNA by using varying annealing temperatures. PCR parameters were as follows: 15 min hot start at 94°C, followed by 35 cycles at 95°C for 30 sec, annealing temperature for 90 sec beginning at 65°C and decreasing by 0.3°C each cycle ending at 54.5°C, and 72°C for 30 sec followed by a final extension of 62°C for 30 min. The second PCR was to label the initial PCR product with the M13 fluorescent dye. The second reaction consisted of 0.3 *μ*L of 10 *μ*mol/L M13, 8 *μ*L Multiplex PCR Master Mix (Qiagen), and 0.6 *μ*L of each specific reverse primer in a 16 *μ*L reaction volume. Parameters for the second PCR included a 15 min hot start at 94°C followed by 25 cycles of 94°C for 30 sec, 55°C for 90 sec, 72°C for 30 sec, followed by a final extension of 62°C for 30 min. Cycling conditions for the two prelabeled primer sets in panel B were, a 15-min hot start at 94°C, 33 cycles of 94°C for 30 sec, 57°C for 90 sec, 72°C for 30 sec, and one cycle of 62°C for 32 min. Fragment analysis was completed on a Perkin Elmer Applied Biosystems 3730 Genetic Analyzer (Carlsbad, California) at the Nevada Genomics Center using 1 *μ*L diluted PCR product added to 19 *μ*L of HiDye Formamide with LIZ (500) molecular marker. ABI GeneMapper software (version 3.7) was used to score alleles.

**Table 2:** Primer information for seven microsatellite loci combined in three multiplex PCR sets

Multiplex panels	Locus-dye	Primer (5′–3′)	Allele size range (bp)	*T*_a_ (°C)
A	Rpi103-FAM	F, TTGAACAGGTATATCTAATAAAGT	135–211	56
		R,TGCTTCCATTTTAATTGTGTC		
	Rpi104-NED	F, CAGGGCAATGTGGAATGTGGA	226–230	62
		R,AGGACCACTCAGGTACAAAATGTTCT		
	RsevMs3-VIC	F, ATGTAAGCAATGCTTGTCC	274–306	55
		R, AAGGACATTGCCACTCAGGC		
B*	Rpi107-FAM	F, GTGGTCTTATTACATTTCTTAC	161–223	57
		R, GCCAGTGAGTGTAGATAGAT		
	Rp193-VIC	F, CCATTTTCTCTCTGATGTGTGT	143–203	44
		R, TGAAGCAGATCACTGGCAAAGC		
C	Rpi101-NED	F, AACGCACAGCAAAGGAGTAA	161–201	62
		R, CAAGGGATGACTTAGAAAGGG		
	RsevF01-FAM	F, GTGGCGTAACATGCCAGTC	163–195	55
		R, CTGTGGATTGAAAGTGTACGC		

Forward (F) and (R) reverse primer sequences, allele size range (bp), and the PCR annealing temperature (T_a_) are provided for each locus.

* Rpi107 and Rp193 were ordered fluorescently labeled and multiplexed together in panel B.

### Spot and color polymorphism assessment

We measured (1) area and perimeter size per spot, (2) total spot number per frog, and (3) color variation to assess phenotypic variation within and among the western Nevada populations (*N*_RFR_ = 61; *N*_FJ_ = 56). No frogs with kandyohi color variation were found. Photographs for 10 of the frogs sampled from each of two sampling localities (RFR and FJ) were chosen to assess total spot number and average spot size (area and perimeter). Only frogs with spots were included in the spot size analysis (Burnsi *N*_FJ_ = 5; Burnsi *N*_RFR_ = 0). If spots were so close together that they were touching and the complete border around each one was not obvious, those spots were counted as one. Images were chosen based on whether all spots could be accurately counted and clearly distinguishable (i.e., frog was being held straight and there was no or limited glare from the camera flash). The spot size data were analyzed using ImageJ version 1.43 (Rasband 1997-2009).

Images were uploaded into ImageJ and converted to eight-bit grayscale pictures. The wand tracing tool and threshold adjustment tool were used to outline and obtain measurements for each spot per frog after establishing the snout–vent length measurement for each frog that was recorded in the field. In ImageJ, measurements to be taken per image were selected under the Analyze tab, in which area and perimeter were selected. The “limit to threshold” option was also chosen so that only what was outlined in the image, using the wand tracing tool, would be measured.

### Statistical analysis

#### Mitochondrial DNA

The program Sequencher (Gene Codes) version 4.2 was used to edit and align raw sequence data obtained from the ABI 3730 DNA Analyzer. We compared the ND1 haplotypes of frogs collected for this study with haplotype data for *L. pipiens* obtained from G.A. Wilson, University of Alberta, Edmonton (Wilson et al. 2008), which included data from Hoffman and Blouin (2004a), in order to assess whether frogs from this study were part of the distinct eastern or western ND1 haplotype clades. The Hoffman and Blouin (2004a) study included 12 leopard frogs sampled from eastern Nevada populations.

#### Microsatellite DNA

We used FSTAT 2.9.3.2 (Goudet 2001) to test for Hardy–Weinberg equilibrium (HWE) for all loci, estimate genetic differentiation between the Truckee and Carson rivers populations (*F*_ST_) (Wright 1969), calculate allelic richness (*R*_S_), the inbreeding coefficient (*F*_IS_) and determine whether linkage disequilibrium among loci was present within populations. We estimated gene diversities (*H*_E_, *H*_O_) using Microsatellite Toolkit in Excel. For the eastern Nevada samples we assessed allelic diversity, richness and private alleles only as these samples were collected from multiple locations (Hitchcock 2001).

We used the program STRUCTURE (version 2.3.2; Pope et al. 2000) to assign individuals into *k* clusters or subpopulations. Individuals sampled from the Truckee and Carson rivers populations and 16 individuals from eastern Nevada were included in this analysis. The underlying assumptions for population modeling in STRUCTURE are that the populations sampled are in HWE and that the loci used to characterize individuals and populations are unlinked and at linkage equilibrium within the populations (Pope et al. 2000). We used an admixture model with parameters set at a 100,000 iteration burn-in period followed by 100,000 Markov Chain Monte Carlo replicates per *k*. The delta *k* (Δ*k*) method developed by Evanno et al. (2005) was used to determine the optimal number of genotype clusters (*k*). This method calculates the largest change in the natural logarithm of the probability of the data (log_e_*P*[*D*]) between each pair of *k* and *k*-1 for all tests of *k*.

We calculated effective population size (*N*_e_) for the Truckee and Carson rivers populations using the linkage disequilibrium method (Hill 1981; Waples 1991; Bartley et al. 1992) in NeEstimator (Peel et al. 2004). The linkage disequilibrium method assesses the amount of linkage disequilibrium (D*) which is Burrow's composite measure of disequilibrium (Campton 1987) and the correlation among alleles at different loci (*r*) (Bartley et al. 1992) in a population. Correlations (*r*) among alleles were estimated by


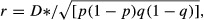


where *p* is the frequency of allele A at locus 1 and *q* is the frequency of allele B at locus 2. The arithmetic mean of all *r*^2^ values is taken across all pairs of loci to obtain a single value that is used to estimate *N*_e_ with the equation, *N*_e_ = [1/[3(*r*^2^-1/*S*)] where *S* is the sample size (Peel et al. 2004).

Genetic bottlenecks for the Carson and Truckee River populations were estimated using the heterozygous excess method, the two phase model (TPM) and single-step mutation model (SMM), and the Wilcoxon sign-rank test in the program BOTTLENECK (version 1.2.0, Cornuet and Luikart 1996). The parameters for the TPM were a 90% stepwise model and 12% variance among multiple steps (Piry et al. 1999). A one-tailed Wilcoxon's signed-rank test was used to test for heterozygote excess (Piry et al. 1999). In addition, each population was checked for a mode shift in allele frequency distribution. In a population at mutation drift equilibrium, the distribution of alleles will be L-shaped (Cornuet and Luikart 1996).

#### Phenotypic variation

An independent *t*-test was used to test for significant differences in total spot number per frog and average size of the spots per frog (as the area and perimeter in centimeters of each spot) between the RFR and FJ populations. The same individuals used for total spot number analysis were used for the spot size analysis. A linear regression analysis using the statistical package *R* was used to test for a significant relationship between frog length and number of spots.

## Results

### Mitochondrial DNA

We successfully sequenced all but one individual from eastern Nevada. A single mitochondrial haplotype (W07) that had been previously described by Hoffman and Blouin (2004a) which is part of the distinct western ND1 haplotype clade was observed in all of the Nevada and the two Idaho individuals sequenced for this study.

### Microsatellites

All loci were in HWE in the Truckee (FJ and CA) and Carson (RFR) rivers populations (*P* = 0.00143, indicative adjusted nominal level [5%], based on 700 randomizations). Although average levels of heterozygosity were higher in the FJ population (*H*_E_ = 0.482, *H*_O_ = 0.477 [FJ]; *H*_E_ = 0.411, *H*_O_ = 0.414 [RFR]; Table 3), they were not statistically significantly higher (analysis of variance [ANOVA], *F* = 0.331, 0.168, *df* = 2, *P* = 0.722, 0.847). Allelic richness was similar for both the RFR (*R*_S_ = 2.24) and FJ (*R*_S_ = 2.25) populations. The CA population had an average heterozygosity of 0.427 and an average allelic richness of 2.10. Allelic richness and diversity for the eastern samples were higher than the two western Nevada populations. However, because the eastern Nevada sample is a composite of frogs from multiple sampling locations, we could not test whether this difference was significant. Private alleles were found primarily in individuals from the eastern basins (alleles in six of eight loci), but were also present in the Truckee and Carson rivers populations (single alleles at four of the eight loci; Table 4).

**Table 3:** Genetic diversity in western Nevada northern leopard frog (*Lithobates pipiens*) populations: observed heterozygosity (*H*_O_), expected heterozygosity (*H*_E_), allelic richness rarified to a sample size of four (*R*_S_), and number of alleles (*A*) for seven microsatellite loci

		Truckee River			Carson River	Eastern Nevada
				
Locus		McR	CA	FJ	RFR	
Rpi103	*N*	4	30	56	61	16
	*H*_O_	0.00	0.46	0.71	0.55	
	*H*_E_	0.00	0.51	0.64	0.48	
	*R*_S_	1.00	2.34	2.96	2.35	
	*A*	1.00	3.00	4.00	3.00	6.00
Rpi104	*H*_O_	1.00	0.17	0.19	0.02	
	*H*_E_	0.57	0.27	0.33	0.02	
	*R*_S_	2.00	1.77	1.85	1.07	2.18
	*A*	2.00	2.00	2.00	2.00	3.00
RsevMs3	*H*_O_	0.00	0.21	0.29	0.53	
	*H*_E_	0.00	0.19	0.31	0.47	
	*R*_S_	1.00	1.61	2.04	1.98	1.53
	*A*	1.00	2.00	4.00	2.00	3.00
Rpi101	*H*_O_	1.00	0.63	0.52	0.56	
	*H*_E_	0.57	0.50	0.49	0.56	
	*R*_S_	2.00	1.99	1.99	2.91	4.61
	*A*	2.00	2.00	2.00	4.00	7.00
RsevF01	*H*_O_	0.00	0.63	0.66	0.51	
	*H*_E_	0.00	0.46	0.51	0.45	
	*R*_S_	1.00	1.98	2.13	2.41	5.41
	*A*	1.00	2.00	3.00	4.00	8.00
Rp193L	*H*_O_	0.50	0.35	0.44	0.53	
	*H*_E_	0.43	0.44	0.45	0.67	
	*R*_S_	2.00	2.32	1.97	3.15	5.45
	*A*	2.00	3.00	2.00	4.00	8.00
Rpi107L	*H*_O_	1.00	0.66	0.53	0.20	
	*H*_E_	0.57	0.62	0.65	0.24	
	*R*_S_	2.00	2.74	2.82	1.85	2.83
	*A*	2.00	3.00	3.00	3.00	5.00
Average *H*_E_		n/a	0.427	0.482	0.411	n/a
Average *H*_O_		n/a	0.444	0.477	0.414	n/a

Population identification is under each designated river sampled.

**Table 4:** Number of alleles per population or sampling location per locus and number of private alleles found in all sampling groups except the captive group (CA)

			Alleles sampled				Private alleles			
				
			Carson River	Truckee River		Eastern Nevada	Carson River	Truckee River		Eastern Nevada
								
	*n* Alleles total	*n* Alleles west	RFR	CA	FJ	RP	RFR	CA	FJ	RP
Rpi101	7	4	4	2	2	7	0	0	0	3
Rpi103	9	5	3	3	4	6	1	0	0	4
Rpi104	2	2	2	2	2	2	0	0	0	0
Rpi107	7	5	3	3	3	5	1	0	0	2
*Rpi108	3	1	1	1	1	3	0	0	0	2
Rp193	10	6	4	3	2	8	1	0	0	4
RsevF01	8	5	4	2	3	8	0	0	0	3
RsevMs3	4	4	2	2	4	3	0	0	1	0

* Rpi108 was excluded from all analysis due to being fixed at a single allele in the western populations. It is included in this table only to indicate the total number of private alleles found in the eastern basin individuals.

**Figure 5:**
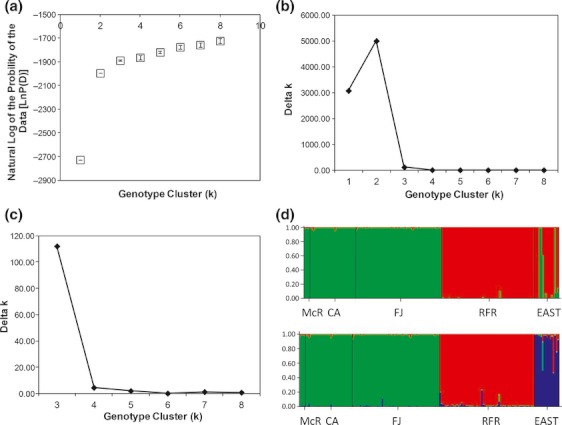
(a) The mean LnP(D) and SD for 10 iterations for each *k* for all individuals included in the study Nevada; (b) delta *k* (Δ*k*) plotted against *k* showing *k* = 2 as the best fit of the data; (c) Δ*k* plotted for *k* = 3–8 only showing that there is some statistical support for *k* = 3; (d) Bayesian genotype clustering results for *k* = 2 and for *k* = 3. The top panel shows the *k* = 2 results. The Truckee River populations are grouped into a single genotype cluster delineated by green (McR, CA [captive population derived from McR] and FJ), the Carson River population groups as a single cluster (red), and the eastern Nevada individuals are a mix of both genotype clusters (red and green). The bottom panel shows the *k* = 3 results. The Truckee and Carson River populations are designated by genotype clusters green and red as in the *k* = 2 analysis, but the eastern Nevada individuals are clearly differentiated in this analysis forming a separate genotype cluster (blue).

All locus combinations in each of the western Nevada populations were tested for linkage disequilibrium in program FSTAT (Goudet 2001). Some loci were linked in the Carson River RFR population but not the Truckee River populations (FJ and CA). This result suggests these loci are not physically linked but appear linked due to the small and isolated nature of the RFR population. As anticipated, the CA and FJ populations in the Truckee River drainage were not genetically differentiated (*F*_ST_ = 0.0124, *P* = 0.0167), whereas the Truckee and Carson River populations were highly differentiated (CA-RFR, *F*_ST_ = 0.425; FJ-RFR, *F*_ST_ = 0.412; *P* = 0.0167). There were no significant *F*_IS_ values for either the Truckee or Carson River populations (*P* = 0.00238, adjusted for multiple comparisons obtained after 700 randomizations, CA, –0.042; FJ, 0.011; RFR, –0.006). Two genotype clusters (*k =* 2) represented the best fit of the data (Fig. 5) with some statistical support for *k* = 3. We include the *k* = 3 results here primarily to show that individuals from the eastern Nevada basins are distinct from those in the west (Fig. 5d).

No evidence was found for a genetic bottleneck under either TPM or SMM for the Truckee River FJ population (*P* = 0.148) or the Carson River RFR population (*P* = 0.531, 0.711, respectively) based on 1000 iterations. However, the FJ population had a shifted mode, which can be a more sensitive test of recent genetic bottlenecks (Piry et al. 1999).

Analysis of microsatellite allele frequencies through NeEstimator (Peel et al. 2004) revealed effective population sizes of 11 (95% CI 8.1–14.9) for RFR, 74.7 (95% CI 32.7–1081.2) for FJ, and 46 (95% CI 32–67) for CA. Despite the fact that this analysis is most precise when at least six loci and 90 individuals are used (Waples 1991; Bartley et al. 1992), these estimates fall between those of other amphibians that are considered widespread and abundant (Zeisset and Beebee 2003; Brede and Beebee 2006). Although the western Nevada leopard frogs have either lower or similar estimates of gene diversity as some widespread species, it is considerably lower than what was found in eastern United States and Ontario, Canada, populations (Hoffman et al. 2004).

### Phenotypic variation

Visual inspection of the color patterns between the frogs sampled on the Truckee and Carson rivers revealed distinct differences. The majority of the RFR frogs sampled (84%; 51 of 61) were green and the other 16% were brown with no burnsi or kandyohi color morphs. Of the 56 frogs sampled from the FJ population, 91% (51 of 56) were brown, with the rest being burnsi color morphs with no green color morphs observed. We found no relationship between frog length and number of spots for frogs sampled in either the Truckee (*P* = 0.185) or Carson (*P* = 0.392) rivers. There was no significant difference between mean number of spots per frog between the FJ (10.1 ± 1.286) and RFR (10.7 ± 1.636) populations (*t* = 0.911; *df* = 18; *P* > 0.05). In addition, there was no significant difference between the mean size of the area of the spots (RFR = 0.401 ± 0.284 cm^2^; FJ = 0.393 ± 0.198 cm^2^; *t* = 0.233; *df* = 206; *P* > 0.5) or the perimeter of the spots (RFR = 2.913 ± 1.329 cm; FJ = 2.851 ± 0.926 cm; *t* = 0.386; *df* = 203; *P* > 0.05) between the natural populations.

## Discussion

The W07 haplotype found in all of the Nevada leopard frogs sampled for this study is widespread throughout the western part of the species range and was the most common haplotype (67%) identified in the eastern Nevada frogs sampled by Hoffman and Blouin (2004a). Two other haplotypes W09 and W16 are known to be unique to Nevada and to date they have only been recorded for populations in eastern Nevada (Hoffman and Blouin 2004a). These haplotypes were not sampled in this study likely due to the small sample size of frogs collected from the eastern portion of the range.

The W07 haplotype is also the most ancestral haplotype in the western ND1 haplotype clade and one of the more common haplotypes found in the western United States as well as western Canada and one population in Ontario (Hoffman and Blouin 2004a). The population in Ontario is thought to be an area where secondary contact between the western and eastern haplotype clades occurred during postglacial expansion of both haplotypes (Hoffman and Blouin 2004a). Although we cannot say definitively that the frogs sampled from the Truckee River are native to the watershed, Bury and Luckenbach (1976) suggest that leopard frogs found east of the Sierra Nevada crest likely represent native stock and not stock derived from frogs translocated from other locations.

The eastern Nevada frogs appear quite distinct from the western populations based on both the Bayesian clustering (*k* = 3) and the private allele analyses, which is not surprising given the geographic separation and the fact that leopard frogs were never widespread in intervening locations in central Nevada. The Carson and Truckee River populations are also highly differentiated (*F*_ST_ 0.41–0.425, Bayesian clustering and color morph analyses), which can be attributed to geographic distance (approximately 90-km straight line distance across various landscape types), and/or contemporary isolation. A high global *F*_ST_ of 0.248 was found among Canadian leopard frog populations (Wilson et al. 2008) separated by at least 250 km. However, some leopard frog populations from the northeastern United States and Ontario, Canada, were less differentiated with a global *F*_ST_ values of 0.034 even though some were separated by 400 km (Hoffman et al. 2004). These northeastern populations are thought to have very large effective population sizes (Hoffman et al. 2004). In addition to the large geographic distance across inhospitable landscapes between populations in the different watersheds in this study, leopard frogs are known to have high site fidelity with 98% returning to their home ranges after being displaced 1– km (Dole 1968) and a maximum recorded dispersal distance of 8 km (reviewed in Smith and Green 2005).

Gene diversity estimates at the microsatellite loci for the two western Nevada populations are within or below the range reported for other *L. pipiens* populations (Hoffman and Blouin 2004a,b; Hoffman et al. 2004; Wilson et al. 2008) and other anurans (Newman and Squire 2001; Brede and Beebee 2004; Funk et al. 2005) including the Dusky gopher frog, *Rana sevosaone*, which is critically endangered (Richter et al. 2009). The range of allelic richness for these populations was also consistent with other *L. pipiens* populations (Hoffman and Blouin 2004b and Wilson et al. 2008), but lower than most populations of another widespread frog, *L. temporaria* (Palo et al. 2004). The higher allelic diversity and presence of private alleles in the eastern Nevada frogs suggests larger overall population sizes and maintenance of higher levels of genetic variation in this area of the range.

As with other western US populations of leopard frogs at the periphery of the species range, the Carson and Truckee River populations have relatively low mean levels of heterozygosity, which has been attributed to historical founder events associated with being peripheral populations (Hoffman and Blouin 2004b). Eckert et al. (2008) concluded from a multistudy review that populations show a decline in genetic diversity and an increase in genetic differentiation at the edges of species ranges. Low genetic diversity compared with eastern Nevada populations and significant differentiation among western Nevada leopard frog populations are consistent with this hypothesis. Lower levels of allelic diversity and heterozygosity in western populations of leopard frog may also be due to fewer refugia available during glacial oscillations resulting in genetic bottlenecks and more severe range contractions over time compared with populations in central and eastern United States. Hoffman and Blouin (2004a) thus suggest that these peripheral leopard frog populations are likely to have always had reduced levels of diversity compared with their interior counterparts.

The contemporary isolation of the Carson and Truckee River populations, however, is of concern as it is likely to lead to erosion of the remaining genetic variation present through population bottlenecks and random genetic drift. Although the *N*_e_ estimate for the FJ population is consistent with other amphibian populations that are considered abundant (Zeisset and Beebee 2003; Brede and Beebee 2006), the Carson River population has a very low *N*_e_ suggesting that this population is extremely isolated. The FJ population has a much larger *N*_e_ than RFR but also showed a shifted mode in allele frequencies. Such shifts in allele frequencies without the detection of significant heterozygosity excess have been reported in other amphibian populations, one of which was attributed to translocations (Beebee and Rowe 2001) and another to founder effects from recolonization (Spear et al. 2006), which may indicate that the FJ population has recently gained new immigrants from unsampled populations or through unknown human mediated movement within the Truckee River basin.

## Conservation Implications

Maintenance of the genetic diversity within the Nevada populations is particularly important due to the large population losses experienced over the past century in this region. Although the majority of private alleles were found in the samples from eastern Nevada, there were also private alleles in both the Carson and Truckee River populations. Despite the fact that we were unable to conduct a population level comparison between populations in eastern versus western Nevada, the results of this study suggest genetic differences that require further examination. Many frog species exhibit metapopulation dynamics and fragmentation and/or degradation of wetlands, and the surrounding landscape can negatively affect the long-term survival of these species (Pope et al. 2000; Semlitsch 2000; Ebisuno and Gentilli 2002). Although we have no evidence of metapopulation dynamics for the leopard frog populations sampled for this study, the lack of significant inbreeding coefficients and the shifted mode results for the FJ Truckee River site suggest that these populations may have been part of larger population networks in the recent past. At least two sites have been occupied on the lower Truckee over the past 20 years (Panik and Barrett 1994; Hitchcock 2001). Hitchcock (2001) found frogs at two sites in the Carson River drainage approximately 20 miles apart, whereas we found only one of those sites occupied for this study. Interestingly, Hitchcock (2001) found each site occupied in alternating years across two sampling seasons.

As peripheral isolates, the Truckee and Carson River populations are likely to lose genetic variation without restoration activities or disappear altogether taking their private alleles and adaptations with them. Lehtinen and Galatowitsch (2001) found that restored wetlands can be valuable for at least some amphibian species, one of which was the northern leopard frog. Restoration of degraded aquatic habitats (Semlitsch 2000) together with creation of continuous corridors that can be used for migration will help to maintain population sizes and encourage gene flow across the landscape, thereby improving the genetic viability of the populations along riverscapes. Genetic differentiation can be overcome through the migration of 1–10 breeding individuals to neighboring populations (Mills and Allendorf 1996), which can decrease the effects of genetic drift and the fixation of deleterious alleles.

The artificial ponds at TNC McR restoration site on the Truckee River were created to enhance population connectivity along the lower reaches of the river. However, the high density of bullfrogs found at most ponds at McR (S. Rogers, pers. obs.) is currently of concern as bullfrogs often inhabit the same ponds that leopard frogs use and also prey upon them in their various life stages (BCMELP 1998; Seburn and Seburn 1998). Declines in leopard frog numbers have been positively correlated with the presence of bullfrogs and carp (*Cyprinus carpio*) (Germaine and Hays 2009).

In the short term, individuals from the Carson River population should be moved to additional locations as a risk-spreading strategy and to preclude losses of genetic variation due to the effects of population isolation. Allendorf and Luikart (2007) suggest that areas being considered for reintroduction should have habitat characteristics that are similar to that of the source population, which may increase the probability that translocated frogs will become established in new locations. Hitchcock (2001) found areas with good-quality habitat in the western watersheds where leopard frogs were present historically, but from which they are currently extirpated. These areas may be vacant due to habitat fragmentation, population isolation, ensuing local extirpation, and the inability of dispersing frogs to reach these areas to recolonize them. However, translocations of frogs simultaneously to multiple suitable habitats in these watersheds could lead to site occupancy sufficient to promote gene flow and increased probability of population persistence. Research into what constitutes effective dispersal corridors is needed. As an interim measure, such sites should be considered for human-mediated translocations.

Another consideration is disease transfer. Chytridiomycosis is caused by the fungal pathogen *Batrachochytrium dendrobatidis* (Bd) and infects keratinized epidermal cells of amphibians such as the oral discs of tadpoles (Fellers et al. 2001; Voyles 2011) and the skin in postmetamorphic individuals (Woodhams et al. 2007). What makes this fungus so dangerous is that virulence varies with species (Woodhams et al. 2007) and some species, including leopard frogs (Woodhams et al. 2008) and bullfrogs (Garner et al. 2006), may harbor the infection without major population die-offs (Voyles 2011). Populations should therefore be screened for Chytridiomycosis prior to translocation using available genetic (PCR) and phenotypic (presence of zoospores) methods (Moore et al. 2011; Tamukai et al. 2011; Voyles 2011).

Additional systematic surveys of the large watersheds of western Nevada (Truckee, Carson, and Walker rivers) is needed to rule out the presence of unsampled populations. If additional populations are found, a landscape genetics approach could be used to test for gene flow and a metapopulation dynamic (Moore et al. 2011). Conservation of this species in an arid environment will entail maintenance of suitable wetland and upland habitats within these watersheds. Efforts by TNC to create interconnected habitat for leopard frogs within the lower Truckee River watershed highlights the challenges presented by invasive nonnative plants and amphibian species. Hitchcock's (2001) analysis suggests that although it is unlikely that a single variable will explain leopard frog declines, aggregates of habitat characteristics can be used to predict where frogs can be found. Sites without frog populations that appear otherwise suitable may indicate that only specific combinations of habitat characteristics can support occupancy by frogs and that long-term occupancy may ultimately be determined at a larger landscape level. The peripheral nature of the populations in western Nevada also underscores the potential additive effects that historical patterns together with contemporary anthropogenic perturbations may have on declines of even widespread species.

## Data Accessibility

DNA microsatellite loci: Genbank accessions *Rana pipiens*: Rpi101 AY157647, Rpi103 AY157648, Rpi 104 AY157649.1, Rpi 107L AY157652; *Rana pretiosa:–* Rp193L Blouin unpublished (Hoffman and Blouin 2004b); *Rana sevosa*:– RsevF01 AY787867, RsevMs3 AY787861. mtDNA ND1 haplotype sequences: Genbank accessions *Rana pipiens* AF548570–AF548613; *Rana pipiens* haplotypes NW1-NW4 were obtained directly from GA Wilson, University of Alberta, Edmonton, Canada; *Rana blairi* (Rbl) AY157644; *Rana sphenocephala* (Rsp) AY157645.
